# Dielectric response of nitrogen in soil amended with chicken litter biochar and urea under *Oryza sativa* L. cultivation

**DOI:** 10.1038/s41598-021-91426-6

**Published:** 2021-06-15

**Authors:** Ali Maru, Osumanu Haruna Ahmed, Walter Charles Primus, Alicia Vanessa Jeffary

**Affiliations:** 1 Institute of Agricultural Research, University of Ghana, P. O. Box 68, Accra, Ghana; 2 Department of Forestry Science, Faculty of Agriculture and Forestry, Universiti Putra Malaysia Bintulu Campus, 97008 Bintulu, Sarawak Malaysia; 3 Institut Ekosains Borneo, Universiti Putra Malaysia Bintulu Sarawak Campus, 97008 Bintulu, Sarawak Malaysia; 4 Institute of Tropical Agriculture and Food Security, Universiti Putra Malaysia, 43400 Serdang, Selangor Malaysia; 5 Institute of Tropical Forestry and Forest Products (INTROP), Universiti Putra Malaysia, 43400 Serdang, Selangor Malaysia; 6 Department of Science and Technology, Universiti Putra Malaysia Bintulu Campus, 97008 Bintulu, Sarawak Malaysia

**Keywords:** Plant sciences, Environmental sciences, Engineering

## Abstract

Unbalanced utilization of nitrogen (N) rice not economically viable neither is this practice environmental friendly. Co-application of biochar and urea could reduce the unbalanced use of this N fertilizer in rice cultivation. Thus, a field study was carried out to: (i) determine the effects of chicken litter biochar and urea fertilization on N concentration in soil solution of a cultivated rice (MR219) using dielectric measurement at a low frequency and (ii) correlate soil dielectric conductivity with rice grain yield at maturity. Dielectric response of the soil samples at 20, 40, 55, and 75 days after transplanting were determined using an inductance–capacitance–resistance meter HIOKI 3522-50 LCR HiTESTER. Selected soil chemical properties and yield were determined using standard procedures. The dielectric conductivity and permittivity of the soil samples measured before transplanting the rice seedlings were higher than those for the soil samples after transplanting. This was due to the inherent nitrogen of the chicken litter biochar and the low nitrogen uptake at the transplanting stage. The soil N response increased with increasing measurement frequency and N concentration. The permittivity of the soil samples was inversely proportional to frequency but directly proportional to N concentration in the soil solution. The estimated contents of N in the soil using the dielectric conductivity approach at 1000 Hz decreased with increasing days of fertilization and the results were similar to those of soil NH_4_^+^ determined using chemical analysis. The conductivity measured within 1000 Hz and 100,000 Hz correlated positively with the rice grain yield suggesting that nitrogen concentration of the soil can be used to estimate grain yield of the cultivated rice plants.

## Introduction

The intensive use of N fertilizers especially in rice production has increased leaching of N fractions such as nitrate and ammonium which have been implicated in eutrophication of water bodies^1^ besides elevating the environmental and health problems^2,3^. Currently, new technologies have been developed in precision agriculture to enable precise use of fertilizers. Among these technologies are Variable Rate Nutrient Application (VRNA), Variable rate irrigation (VRI), Controlled Traffic Farming (CTF), and management zones to manage spatial and temporal variability in farming systems.

These technologies were developed to ensure that inorganic fertilizers and manure are used based on plant nutrient requirement (Site specific management). However, there is dearth of information on innovations for measuring N in rice fields. The traditional methods of determining plant nutrients in soils are destructive, laborious, time consuming, and expensive^4^ particularly when a need for monitoring N concentration in the soil solution arises. Previously, there was a wide adoption of sensors to measure the chemical properties of a medium through dielectric response. Additionally, bulk soil permittivity measurement was used to estimate volumetric water content^5,6^. However, these methods cannot be used in lowland rice fields where the soils are saturated with water. Time domain reflectometry (TDR) techniques have been also used to estimate changes in nitrate (NO_3_^−^) concentration in soil solution^7,8^ but these techniques are unsuitable for field experiments because this approach expensive and inaccurate^9^.

Electrical conductivity of soil on permittivity measurement is negligible at high frequencies (above 50 MHz) but it becomes stronger with decreasing frequency measurement^10^, making capacitance sensors response to soil salinity less effective^10^. Soil electrical conductivity strengthens as frequency decreases, suggests that there is a potential to measure ionic concentration in soil solution at multiple frequencies through analysis of spectral data patterns. However, little work has been done in estimating ionic concentration of soil water. To date, there is dearth of information on estimating ionic concentration of soil water in rice fields using parallel plate capacitor at low frequency. Therefore, the objectives of this study were to:

1. Determine the effects of co-use of chicken biochar and only urea on nitrogen concentration in a tropical acid soil cultivated with MR219 rice variety using low frequency dielectric measurement.
2. Correlate soil dielectric conductivity of the four growing stages (20, 40, 55, and 75 days after transplanting) of MR219 rice variety with grain yield at maturity.

## Materials and methods

A field study was conducted on Nyalau series (Typic Paleudults) at Universiti Putra Malaysia Bintulu Campus, Malaysia. In this study, the samples collected for analysis were all produced and analyzed within Universiti Putra Malaysia and all legislation of Universiti Putra Malaysia was complied with. In this present study Randomized Complete Block Design with four blocks was adopted. Each plot size was 2 m (length) × 2 m (breadth). The distance between the plots was 1 m and that between the blocks was 3 m. The treatments used in this study were:

- T1:Soil only
- T2:Soil + urea (46% N) only
- T3:Soil + chiken litter biochar + 100% urea-N
- T5:Soil + chiken litter biochar + 75% urea-N
- T6:Soil + chiken litter biochar + 50% urea-N
- T7:Soil + chiken litter biochar + 25% urea-N
- T8:Soil + chiken litter biochar only

### Chemical composition of chicken litter biochar

The organic amendment used in this study was an Australian biochar and according to Australia Certified Organic Standard 2010, the heavy metals of this biochar is below the permitted threshold (Table 1). The biochar rates were based on 5 t ha^−1^ (Maru et al., 2015) which is equivalent to 2000 g plot^−1^. The 2000 g were spread on the soil surface of the experimental plots and afterwards, they were mixed with the soil a day before transplanting. The fertilizers used for the MR 219 rice variety were based on the existing fertilization programme^11^ (Table 2).

**Table 1:** Selected chemical properties of BlackEarth chicken litter biochar.

Macro nutrients			Micro nutrients		
pH	8.5	Av. particle size	0.5–2 mm		
%		mg kg^−1^			
Total carbon	63.7	Silica	2.3	Magnesium oxide	6.7
Fixed carbon	61.2	Alumina	1.5	Arsenic	2.1
Nitrogen	2.8	Potassium oxide	16.3	Cadmium	0.7
Phosphate	2.6	Boron	62	Chromium	9.6
Potassium	3.9	Copper	167	Mercury	0.06
Calcium	5.9	Manganese	1130	Nickel	14
Sulphur	0.59	Zinc	856	Lead	12

*Source*: BlackEarth Company in North of Bendigo Victoria, Australia.

**Table 2:** Chicken litter biochar rate and fertilization schedule for the field study.

Plant growth stages		Early tillering growth	Active growth	Formation of stalk	Grain filling
Days after transplanting		15 to 20	35 to 40	50 to 55	70 to 75
Treatments	Biochar rates				
g plot^−1^					
T1	0	0	0	0	0
T2	0	Mix A2	40	Mix B1	Mix B1
T4	2000	55 urea only	40	18 urea only	18 urea only
T5	2000	40.3 urea only	30	14 urea only	14 urea only
T6	2000	27.5 urea only	20	9 urea only	9 urea only
T7	2000	13.8 urea only	10	5 urea only	5 urea only
T8	2000	0	0	0	0

Mix A2 = (55 g Urea + 50 g TSP + 24 g MOP).

Mix B2 = (18.3 g Urea + 18.7 g TSP + 19.8 g MOP + 1.4 MgO).

TSP—Triple superphosphate; MOP—Muriate of potash; MgO—Magnesium oxide.

Standard N solutions ranging from 0 to 10% N was prepared using urea and distilled water. Soil samples was collected at four growth stages of the cultivated MR219 rice plants, namely early tillering growth (20 days after transplanting), active growth (40 days after transplanting), stalk formation (55 days after transplanting), and grain formation stages (75 days after transplanting). Afterwards, the plastic vials were tightly closed and transferred to the laboratory for immediate analysis.

### Dielectric measurement

**Figure 1:**
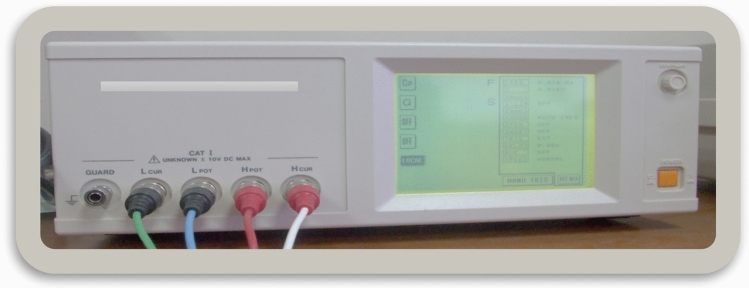
Inductance–capacitance–resistance (ICR) meter HIOKI 3522-50 LCR HiTESTER.

Dielectric materials are important medium for electronic circuit. Depending on the dielectric properties of materials, electronic circuits are built on either high or lower frequency. Dielectric property is a characteristic of plant materials because of the structure of the biomaterials and the high water content. A dielectric material is an electrically insulating material which polarizes in an electric field and it provide valuable data on storing and releasing electric and magnetic fields in materials and the potential of using such materials. The permittivity is used an index of polarizability of a material. The practical part of permittivity is dielectric constant. Dielectric response of the soil samples was measured using an inductance–capacitance–resistance (ICR) meter HIOKI 3522-50 LCR HiTESTER (Fig. 1).

**Figure 2:**
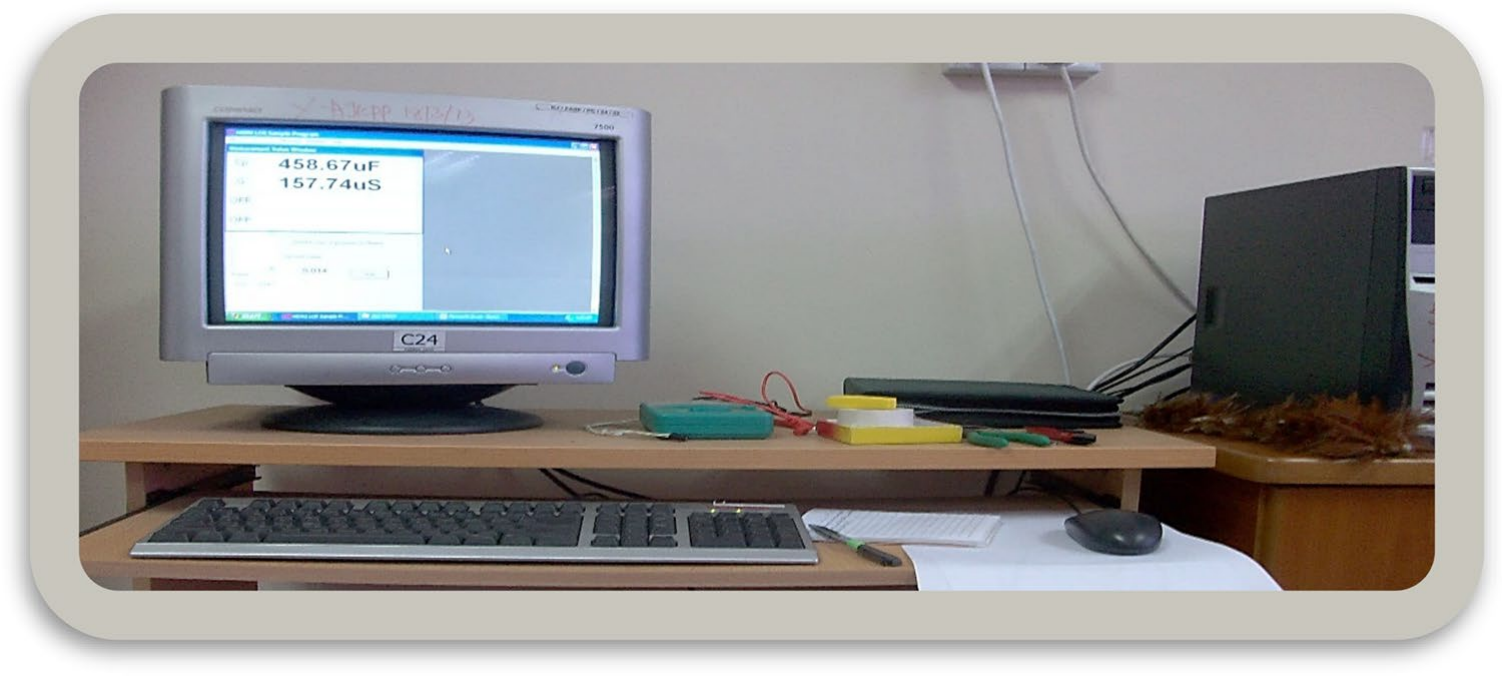
Monitor that received the data during measurement.

**Figure 3:**
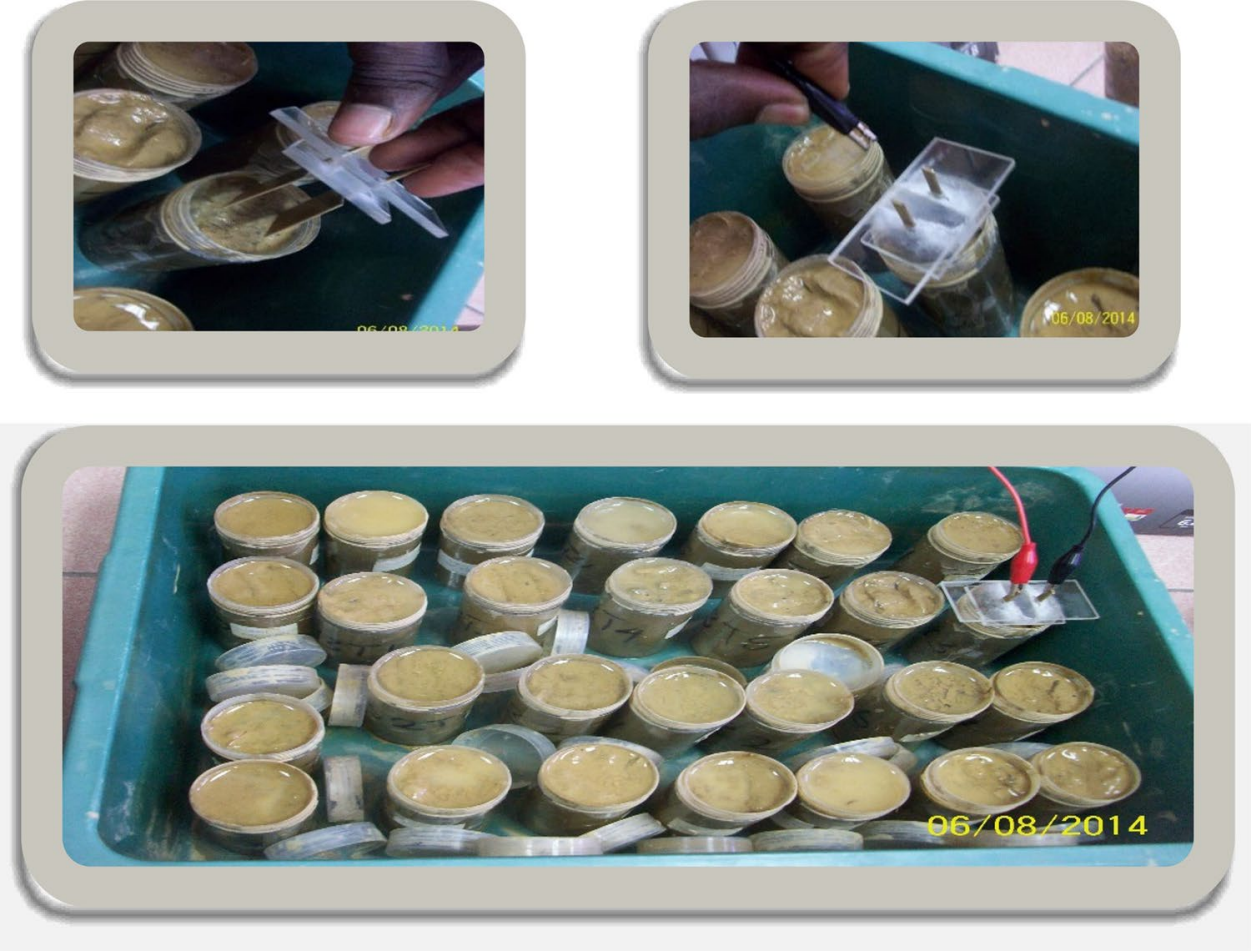
Parallel plate capacitor being inserted into the soil samples in a plastic vail.

A parallel plate capacitor with two conducting plates with an area of 7.5 $\times {10}^{-4}$ m^2^ facing each other at 0.015 m apart was connected to ICR meter and the ICR meter was connected to a computer which received the data. Afterwards, the parallel plate capacitor was inserted into the soil in a plastic vial after which the dielectric response was measured within 0.01 Hz and 100 kHz at 50 points (Figs. 2, 3). Data obtained were the real part of capacitance (*C*′) and conductance (*G*). Thereafter, the permittivity and imaginary capacitance of the soil samples were calculated.

### Data transformation

The data for conductance (*G*) were substituted into the Eq. (1) to get the imaginary capacitance (*C*″).

1 $$C^{\prime \prime } = \frac{G}{\omega }$$

where $$\omega$$ = $$2\pi \times Frequency$$.

The dielectric $\mathrm{conductivity} \left(\sigma \right)$ was calculated using Eq. (2)

2 $$\mathrm{Conductivity} \left(\sigma \right)=G\frac{d}{A}$$

where d is the distance between the two antenna plates of the electrolyte parallel plate capacitor facing each other (1.5 cm) and *A* is the area of antenna plates is 7.5 cm^2^. The permittivity at the real ($\varepsilon {^{\prime}})$ and imaginary ($\varepsilon {^{\prime \prime}})$ were calculated, thus $\varepsilon {^{\prime}}$ is dielectric constant describes polarization ability of the material and ${\varepsilon }{{^{\prime \prime}}}$ is the dielectric loss factor that describes the ability of the material to dissipate energy.

$${\text{Real permitivity }}\left( {\varepsilon^{\prime } } \right) = \frac{{C^{\prime } }}{{C_{0} }}{\text{and }}$$

$${\text{Imaginary permitivity }}\left( {\varepsilon^{\prime \prime } } \right) = \frac{{C^{\prime \prime } }}{{C_{0} }}$$

where $${ C}_{0}= {\varepsilon }_{0}\frac{d}{A} and {\varepsilon }_{0}\mathrm{ is a constant }$$.

Dielectric response of the soil samples was measured using an inductance–capacitance–resistance (LCR) meter HIOKI 3522-50 LCR HiTESTER. A parallel plate capacitor with two conducting plates with an area of 7.5 × 10^−4^ m^2^ facing each other at 0.015 m apart was connected to the LCR meter. The LCR meter was connected to a computer which received the data. A parallel plate capacitor was inserted in the soil in the plastic vials and the dielectric response was measured within 0.01 Hz and 100 kHz at 50 points. Data obtained were the real part of capacitance (C′) and conductance (*G*) after which permittivity and conductivity were calculated.

### Determination of rice grain yield

The rice plants were harvested at different maturity days because of the different treatment effects on the rice grain ripening. At harvest, number of tillers and number of panicles of the rice plants were determined. Ten panicles for each plot were used to determine grain filling and the rice plants’ grain yield. Matsushirna and Tanaka^12^ method (Eq. 3) was used to calculate the total rice yield for the treatments.

3 $$Yield=\frac{\mathrm{weight\, of }1000\,\mathrm{grain}*\mathrm{spikelet}*\mathrm{\%total \,grain \,filled}}{10,000\mathrm{ \,m}2*1000}$$

where the area for 1 hectare = 10,000 m^2^ was used to enable the yield to be expressed in hectare and the 1000 was used to get the dry weight of 1 grain.

### Soil and plants chemical analysis

Following oven drying (60 degrees C until constant weight was obtained) and grinding of the harvested rice plants’ shoot, total N was determined using Kjeldhal method^13^ and inorganic N (NO_3_^−^ and NH_4_^+^) was determined using the method described by^14^. The nutrient contents were multiplied by the dry matter of the rice plants to represent the nutrients taken up by the rice plants.

### Statistical analysis

Analysis of variance (ANOVA) was used to determine treatment effects on soil and above ground biomass whereas treatments means were compared using Tukey’s Test^15^. Pearson correlation analysis was used to correlate variables using the Statistical Analysis Software version 9.3.

## Results and discussion

### Dielectric conductivity of different nitrogen concentrations measured within 0.01 Hz to 100 kHz

**Figure 4:**
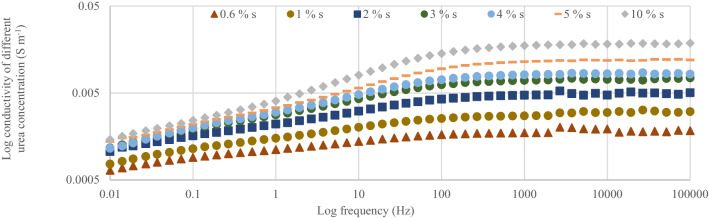
Conductivity of N solution (0.6%, 0.8%, 1.0%, 2.0%, 3.0%, 4.0%, 5.0%, and 10.0%) at a frequency range of 0.01 Hz to 100 kHz.

**Figure 5:**
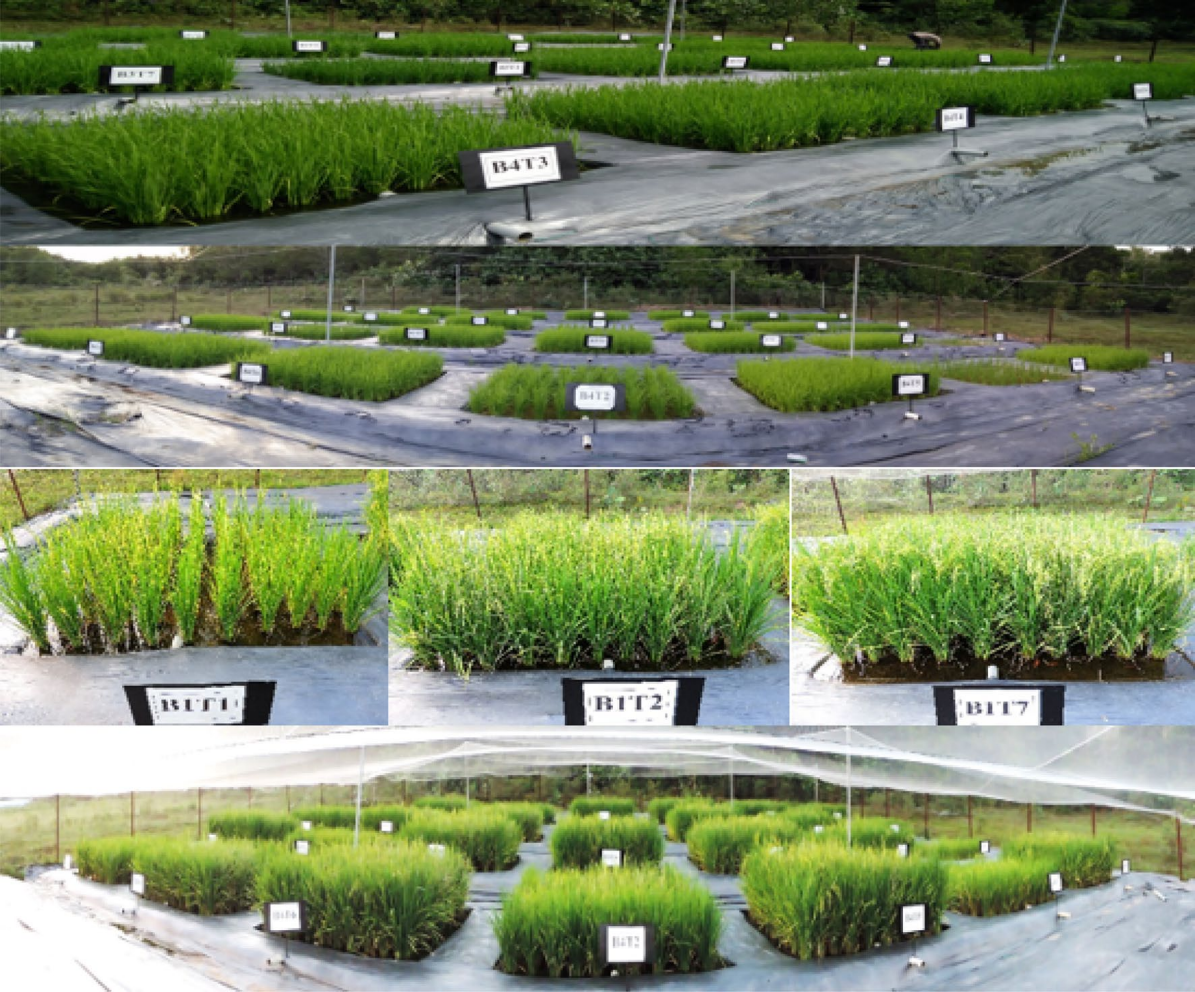
Growth of rice plants at eighty days after transplanting.

**Figure 6:**
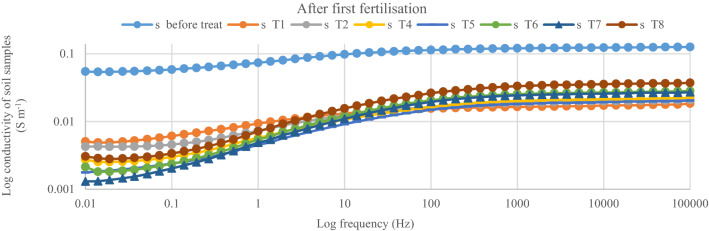
Conductivity of soil samples after first fertilization (20 days after transplanting) within 0.01 Hz and 100 kHz.

**Figure 7:**
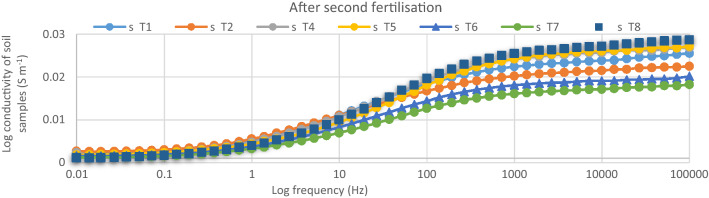
Conductivity of soil samples after second fertilization (40 days after transplanting) within 0.01 Hz and 100 kHz.

**Figure 8:**
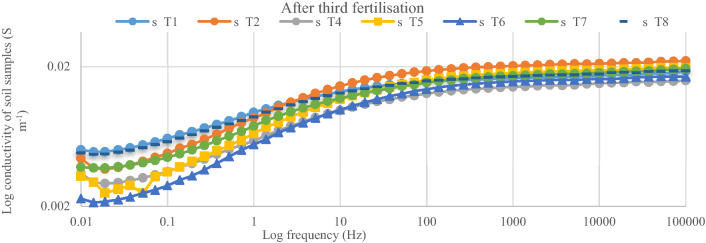
Conductivity of soil samples after third fertilization (55 days after transplanting) within 0.01 Hz and 100 kHz.

**Figure 9:**
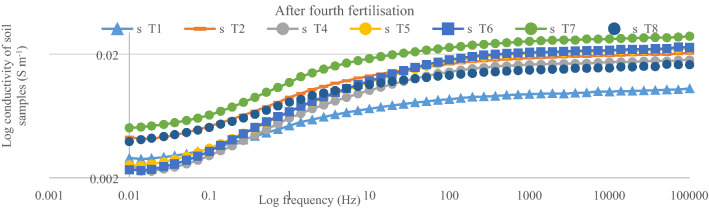
Conductivity of soil samples after fourth fertilization (75 days after transplanting) within 0.01 Hz and 100 kHz.

After the first fertilization, the dielectric conductivity (DC) pattern of the spectral responses for the soil samples within 1000 Hz and 100 kHz was in the order of < T5 ≤ T2 < T4 < T7 ≤ T6 < T8 (Fig. 4). The conductivity of the soil only (T1) was the lowest and this explains why the rice plants under T1 got stunted. The soil conductivity with T5 and T2 were similar despite T2 having 100% urea as the positive control (Fig. 5). This observation is related to lower tillering of the rice plants under T2 compared with T5 which had 75%urea. The lower tillering under T2 significantly reduced N uptake compared with T5 (Fig. 5). Also, the soil conductivity under T4 was higher than with T2 although T4 also had 100% urea but higher tillering, suggesting that the chicken litter biochar can be used to improve N in the soil solution^16^. Nitrogen in the plots with T8 showed the highest conductivity response (Fig. 6) in spite of having only chicken litter biochar. Similar magnitude, shape, and pattern of the spectral responses of the soil samples in the second, third, and fourth urea applications were observed. After the second fertilization, the soil conductivity with T1 was higher than that of T2 (Fig. 7). However, the conductivity decreased after the third and fourth fertilizations (Figs. 8, 9). The Soil conductivity increased during the second and third fertilizations because of the poor nutrient uptake by the stunted rice plants. The depletion of N after the fourth fertilization (Fig. 9) was due to leaching and volatilization of N. Although the conductivity under T2 was the highest during the third fertilization, there were no significant responses among all treatments due to higher N uptake by the rice plants at the tillering stage^17,18^. The soil conductivity in the plots with T2 was the highest due to higher fertilization coupled with less N use efficiency of the rice plants (Fig. 8)**.**

**Figure 10:**
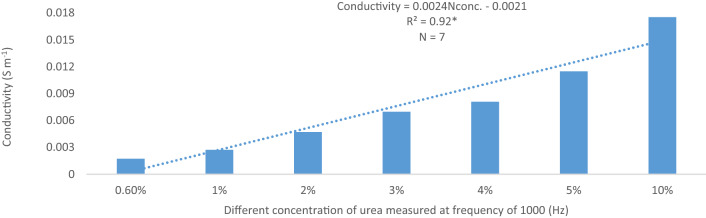
Conductivity of N solution (0.6%, 0.8%, 1.0%, 2.0%, 3.0%, 4.0%, 5.0%, and 10.0%) within 0.01 Hz and 100 kHz.

The conductivity of the standard N solution indicates that dielectric response of N is more sensitive at Frequency within 1000 Hz and 100 000 Hz (Fig. 10). This suggests that, magnitude, shape, and pattern of the spectral responses of the soil samples to conductivity at this frequency was due to N concentration of the soil solution (Figs. 6, 7, 8, 9). However, the conductivity of the standard N solution within 0.01 Hz and 1 Hz was less sensitivity to dielectric response (Fig. 10) compared with that of the soil samples (Figs. 6, 7, 8, 9). This suggests that other elements in the soil samples were more sensitive to conductivity within 0.01 Hz and 1 Hz than N.

The dielectric responses (Fig. 2) for the soil samples (first fertilization) demonstrated the following spectral responses: (i) unsaturated soil (soil sample before treatment) and (ii) saturated soil (soil during cultivation). These results indicate that the moisture content of the soil samples affected the dielectric response^19^. The higher conductivity of the soil samples before treatments application was due to the higher concentration of N in the soil solution because these samples had lower moisture. The waterlogged soil samples demonstrated lower conductivity not only because of lower concentration of N but also because of excess soil water. Moreover, excess free water molecules might have tightly bonded with clay and NH_4_^+^ ions resulting in the reduced conductivity (Figs. 6, 7, 8, 9).

The relationship between conductivity at 1000 Hz and urea concentration of the standard N solution was determined (Fig. 10). There was a linear relationship between conductivity at 1000 Hz and urea concentration of the standard N solution suggesting that the conductivity increased with increasing urea concentration (0.6%, 1%, 2%, 3%, 4%, 5%, and 10%) (Fig. 10). The equation in Fig. 10 was used to estimate N concentrations of the soil samples measured at 1000 Hz (Table 3) and these concentrations were compared with the N concentrations at harvest (Table 4).

**Table 3:** Estimated nitrogen concentrations of the soil samples measured using dielectric conductivity at 1000 Hz.

Nitrogen concentration Days after transplanting		T1	T2	T4	T5	T6	T7	T8
		%						
20 days of transplanting		10.22	9.47	9.52	10.79	9.82	7.55	8.32
35 days of transplanting		10.21	9.29	11.50	10.94	8.46	8.39	8.32
55 days of transplanting		7.73	9.27	6.81	8.33	7.35	8.01	7.92
75 days of transplanting		4.82	8.60	7.71	9.29	9.47	11.45	7.06

**Table 4:** Soil nitrogen concentrations measured after harvesting using chemical analysis method in the first planting cycle.

Treatment	Total N (%)	Available NO_3_^−^ (mg kg^−1^)	Exchangeable NH_4_^+^ (mg kg^−1^)
Initial	0.05b ± 0.007	1.05a ± 0.35	1.58abc ± 0.18
T1	0.04b ± 0.008	0.88a ± 0.18	1.93d ± 0.18
T2	0.05b ± 0.013	1.05a ± 0.20	3.33d ± 0.34
T4	0.11a ± 0.013	2.10a ± 0.29	3.68 cd ± 0.18
T5	0.11a ± 0.013	1.75a ± 0.20	2.80a ± 0.08
T6	0.09ab ± 0.013	1.75a ± 0.20	2.98bc ± 0.15
T7	0.09ab ± 0.007	1.93a ± 0.34	2.98abc ± 0.18
T8	0.07b ± 0.008	1.40a ± 0.29	2.28ab ± 0.22

Different letters within a row indicate significant difference between means of four replicates ± standard error using Tukey’s test at *P* ≤ 0.05.

### Estimated nitrogen concentrations of soil samples measured using dielectric conductivity

The estimated N concentrations of the soil samples measured using dielectric conductivity at 1000 Hz demonstrated that, the soil N concentration decreased with increasing days of fertilization until day 55, after which an increase in N concentration was observed. This was because the N uptake increases with increasing plant growth and the N requirement after 55 days was higher due to booting and panicle heading of the rice plants. After the first fertilization, the N concentration of the soil solution for T2 was higher than those of T7 and T8 (Table 3). The rice plants growth and N uptake with T4, T5, and T6 were higher than with T2 (Table 3), confirming the ability of the chicken litter biochar to increase soil nutrient availability^20^. A similar soil N concentration was observed after the second fertilization except for T6 because of higher N uptake by the rice plants to support initial growth (Table 3). After the third fertilization, N concentration under T2 was higher than with T4, T5, T6, T7, and T8 (Table 3) because of the poor rice plants’ growth and N use efficiency. However, after the fourth fertilization, N concentration under T2 was lower than those with T5, T6, and T7 due to leaching of N, indicating that the chicken litter biochar enhances retention of soil nutrients.

The N concentration in the soil only (T1) was higher after the first and second fertilizations due to the poor rice plants’ growth and N use inefficiency although the N concentration decreased rapidly after the third and fourth fertilizations probably because of leaching of N (due to high rainfall). The estimated N concentration of the soil samples measured using dielectric conductivity at a 1000 Hz was similar to that of the exchangeable NH_4_^+^ determined using the traditional chemical analysis approach. However, the exchangeable NH_4_^+^ (Tables 4, 5) was lower than the estimated N concentration using dielectric approach (Table 3) because the soil samples for dielectric measurement were analyzed immediately after sampling. Furthermore, because the soil samples for chemical analysis were air dried and ground to pass a 2 mm sieve some of the N (exchangeable NH_4_^+^) might have been be lost during this process.

**Table 5:** Soil nitrogen concentrations measured after harvesting using chemical analysis method in the second planting cycle.

Treatment	Total N (%)	Available NO_3_^−^ (mg kg^−1^)	Exchangeable NH_4_^+^ (mg kg^−1^)
T1	0.12a ± 0.01	2.37a ± 0.22	2.68b ± 0.25
T2	0.09a ± 0.02	2.34a ± 0.23	3.40ab ± 0.20
T4	0.13a ± 0.01	3.04a ± 0.62	4.44a ± 0.47
T5	0.12a ± 0.01	1.87a ± 0.23	4.67a ± 0.23
T6	0.14a ± 0.01	2.10a ± 0.01	4.67a ± 0.23
T7	0.12a ± 0.01	2.20a ± 0.10	4.44a ± 0.23
T8	0.09a ± 0.02	3.27a ± 0.84	3.50ab ± 0.01

Different letters within a row indicate significant difference between means of four replicates ± standard error using Tukey’s test at *P* ≤ 0.05.

### Correlation among nitrogen fertilization, log real conductivity at different frequencies and grain yield

Urea-N, log conductivity (0.01, 0.1, 1, 10, 100, 1000, 10,000, and 100,000 Hz), and grain yield were correlated (Table 6). Urea-N and conductivity at 1000, 10,000, and 100,000 Hz correlated positively with the rice grain yield suggesting that the log real conductivity measured within 1000 Hz to 100,000 Hz had a positive association with the rice grain yield. This confirms that, dielectric responses of N concentration in lowland rice fields can be measured at 1000 Hz and the log real conductivity of this measurement is closely related to rice grain yield.

**Table 6:** Correlation among nitrogen fertilization, log real conductivity at different frequencies and grain yield.

	Nap	C (F 0.01)	C (F 0.1)	C (F 1)	C (F10)	C (F 100)	C (F 1000)	C (F 10,000)	C (F100000)
C (F 0.01)	0.42								
	0.48								
C (F 0.1)	0.26	0.88*							
	0.68	0.05							
C (F1)	0.51	0.88*	0.96*						
	0.39	0.05	0.01						
C (F 10)	0.60	0.85	0.91*	0.99*					
	0.29	0.06	0.03	0.002					
C (F 100)	0.73	0.80	0.81	0.93*	0.98*				
	0.16	0.11	0.10	0.02	0.004				
C (F 1000)	0.79	0.73	0.74	0.90*	0.95*	0.99*			
	0.11	0.16	0.15	0.04	0.01	0.0007			
C (F 10,000)	0.79	0.73	0.75	0.90*	0.95*	0.99*	1.00*		
	0.11	0.16	0.15	0.04	0.01	0.0008	< .0001		
C (F 100,000)	0.79	0.75	0.76	0.91*	0.96*	0.99*	1.00*	1.00*	
	0.11	0.15	0.14	0.03	0.01	0.0006	< .0001	< .0001	
	0.86	0.58	0.62	0.79	0.81	0.86	0.90*	0.91*	0.90*
Rice yield	0.06	0.31	0.26	0.11	0.10	0.06	0.04	0.03	0.04

*Show correlation with yield at *P* ≤ 0.05. Where Nap = Urea applied during field study, C = Log conductivity, and F = Frequency at which soil samples were measured.

## Conclusions

The dielectric conductivity and permitivity of the soil samples measured before transplanting the rice seedlings were higher than those for the soil samples after transplanting. This was because of the inherent nitrogen of the chicken litter biochar and the low nitrogen uptake at the transplanting stage. The soil N response increased with increasing measurement frequency and N concentration. The permitivity of the soil samples was inversely proportional to frequency but directly proportional to N concentration in the soil solution. The estimated contents of N in the soil using the dielectric conductivity approach at 1000 Hz decreased with increasing days of fertilization and the results were similar to those of soil NH_4_^+^ determined using chemical analysis. However, within 1000 Hz and 100,000 Hz, the N concentration in soil solution was more sensitive to conductivity than within 0.01 Hz and100 Hz. The conductivity measured within 1000 Hz and 100,000 Hz correlated positively with the rice grain yield suggesting that nitrogen concentration of the soil can be used to estimate grain yield of the cultivated rice plants.
